# Glioblastoma multiforme presenting as postpartum depression: a case report

**DOI:** 10.1186/s13256-018-1909-3

**Published:** 2018-12-20

**Authors:** Johannes Petzold, Emanuel Severus, Shirin Meyer, Michael Bauer, Dirk Daubner, Dietmar Krex, Tareq A. Juratli

**Affiliations:** 1 Department of Psychiatry and Psychotherapy, Carl Gustav Carus University Hospital, TU Dresden, Dresden, Germany; 2 Institute of Neuroradiology, Carl Gustav Carus University Hospital, TU Dresden, Dresden, Germany; 3 Department of Neurosurgery, Carl Gustav Carus University Hospital, TU Dresden, Dresden, Germany

**Keywords:** Case reports, Depression, Postpartum period, Cognitive dysfunction, Psychopathology, Diagnosis, Magnetic resonance imaging, Glioblastoma, Isocitrate dehydrogenase, Mutation

## Abstract

**Background:**

Alterations of mental status are characteristic of psychiatric disorders but may also result from a multitude of organic causes. Generally, physical examination and blood analysis are a part of basic psychiatric differential diagnostics, whereas more sophisticated procedures (for example, brain imaging) are applied only in cases with pathologic diagnostic findings. Our report challenges this approach by describing a case of glioblastoma multiforme presenting as postpartum depression without abnormalities in basic differential diagnostics.

**Case presentation:**

A 28-year-old white woman who had been in outpatient treatment for postpartum depression was taken to the psychiatric emergency room. The psychopathological assessment, however, showed mild disorientation and severe deficits of long-term memory. Moreover, she complained of stabbing, bilateral headaches, but results of her physical examination and blood analysis were unremarkable. Magnetic resonance imaging of the brain was performed, which showed a contrast-enhanced mass lesion in the left frontal lobe. The patient underwent urgent tumor resection, and histologic results revealed an *IDH*-mutant glioblastoma multiforme. The patient was discharged with a substantially improved psychopathology and without neurological deficits.

**Conclusions:**

This report adds to the evidence that postpartum depression may have organic causes in some cases, a fact that needs to be considered in the clinical setting. Atypical neurocognitive findings in a psychiatric interview may alone justify brain imaging, despite normal physical examination and blood analysis results.

## Background

Alterations of mental status are characteristic of psychiatric disorders but may also result from various medical diseases, such as metabolic disorders, infections, and brain lesions [1]. Therefore, a comprehensive differential diagnostic procedure should be an essential part of every psychiatric assessment and should include neuroimaging in unusual cases. In this report, we present a case of a pregnant woman with unremarkable diagnostic assessments during her pregnancy. After a complicated birth, the patient showed an acute onset of neurocognitive symptoms that were initially interpreted as postpartum depression. However, further diagnostic assessment revealed a large frontal brain tumor that was resected and proved to be a glioblastoma multiforme (GBM). To date, only a few cases of a brain tumor mimicking postpartum depression have been described [2, 3].

## Case presentation

A 28-year-old white woman checked herself into an outpatient clinic of psychosomatic medicine and psychotherapy (PSO) for the first time. She reported having a vacuum-assisted child delivery 6 weeks prior, during which significant blood loss led to the surgical removal of her placenta. Since then, she could not bond with her baby and had been experiencing feelings of emptiness as well as a decrease in energy and general happiness. In addition, she had withdrawn from social activities. Later, she began experiencing migraines. During her visit, she was short-spoken, emotionless, and gave conflicting responses to simple questions. When asked about her mood, she indicated that she was feeling very relaxed.

Three days later, the patient was referred to the psychiatric emergency room for ambiguous psychopathology and progressive headaches. Upon arrival, she was not able to explain why she was in treatment at the outpatient clinic for PSO. The patient’s mother added that her daughter had barely spoken or answered questions over the past 2 days. The patient later reported that she had stopped breastfeeding owing to insufficient lactation while also experiencing sharp, bilateral headaches (intensity 7, 0 = no pain, 10 = unimaginable pain) without noticeable triggers. These headaches lasted about 10 minutes, occurred several times per day, and had appeared for the first time 6 days before her second visit. She also described having nausea and flashes of light in her left eye. Painkillers did not relieve the migraines during the day, but she slept comfortably and pain-free at night. She did not take any other drugs, and she had never before had mental disorders or other relevant diseases. Her micturition and defecation were normal. She did not have night sweats, fever, or weight loss. Her social network was supportive. There was nothing of note in her family medical history. A psychopathological assessment demonstrated mild disorientation (date indeterminable, wrong month), a severely impaired capacity to concentrate (not able to solve easy arithmetic problems), deficits in long-term memory, poverty of speech (sparse replies to questions, sometimes delayed or unanswered), a flat affect, and feelings of helplessness. There were no indications of rumination, incoherence, delusions, perceptual disturbances, movement disorders, or aggressive thoughts toward herself, her baby, or others. The targeted physical examination did not show pathologies.

On the basis of the acute onset of these symptoms after a complicated birth and a normal physical examination, she could have been diagnosed with severe postpartum depression. Nevertheless, the sudden onset of severe headaches with flashes of light, disorientation, and long-term memory impairment raised suspicions. Therefore, a neurologist was consulted. Blood analysis results ruled out the possibility of a cerebral venous sinus thrombosis and, although it showed mild anemia and leukocytosis, revealed normal C-reactive protein and D-dimer levels. In response to the recurrent headaches that appeared to be depression-related, recommendations were made for the patient to take a fixed intake of nonsteroidal anti-inflammatory drugs for the next few days.

**Fig. 1:**
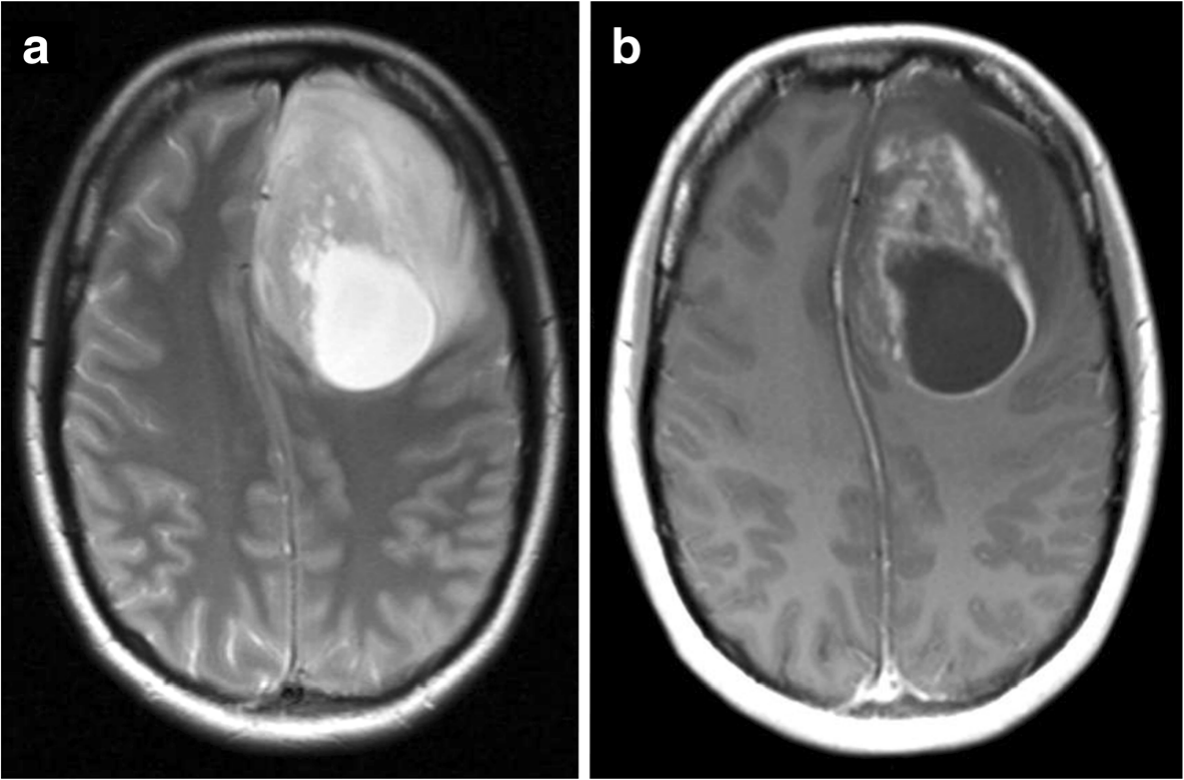
A heterogeneous tumor (65 × 50 mm) in the left frontal lobe with solid and cystic segments partly showing strong, irregular, and ring-shaped contrast enhancement. **a** T2-weighted image. **b** T1-weighted image with contrast agent

As a result of the conspicuous psychopathology, the patient was advised to undergo magnetic resonance imaging (MRI), but she ultimately declined. Two days later, the patient was admitted for acute right-sided hemiparesis. A brain MRI scan displayed a contrast-enhanced mass lesion in the left frontal lobe (Fig. 1). The patient underwent urgent tumor resection that revealed a GBM harboring an isocitrate dehydrogenase 1 mutation (*IDH1*). After 10 days, the patient was discharged with a substantially improved psychopathology and without neurological deficits. The subsequent concordant radiochemotherapy, which was initiated 2 weeks after discharge, was tolerated very well. Owing to chemotherapy, the patient was not able to breastfeed her baby. At the last follow-up, almost 3 years after tumor resection, the patient was in excellent mental and physical condition with no evidence of tumor recurrence.

## Discussion

We describe a case of a young woman who showed an acute onset of neurocognitive symptoms shortly after giving birth to a healthy baby. The symptoms were initially interpreted as postpartum depression. However, further investigations revealed an intracerebral neoplasm that was resected and histologically confirmed as an *IDH*-mutant GBM. Somatic mutations of the isocitrate dehydrogenase 1 and 2 genes (*IDH1* and *IDH2*) are frequent and early events in the pathogenesis of low-grade gliomas as well as in a small subset of GBMs [4, 5]. Patients with *IDH*-mutant tumors have an improved overall survival [4] and less frequent neurocognitive impairments [6], compared with patients with *IDH* wild-type gliomas. Notwithstanding, the patient in our case study showed major neurocognitive deficits that were probably due to the large size of the tumor at diagnosis and the subsequent midline shifting. Previous reports have found some evidence for an increased rate of tumor growth and malignant transformation of gliomas during pregnancy [7]. One could speculate that the rapid development of neurocognitive deficits in our patient was due to a rapid progression of tumor growth in the last weeks of pregnancy, although *IDH*-mutant GBMs typically grow slowly [8]. Notably, it has been hypothesized that the negative interaction between pregnancy and glioma growth is associated with major changes in maternal hormones [9]. The latter assumption is substantiated by the observation that patients who develop breast cancer before the age of 45 have a significantly higher risk of developing GBM, presumably owing to a higher estrogen level in the years before menopause [10, 11]. Noteworthy, the influence of maternal hormones on tumor development is well established for breast cancer [12]. Beyond the hypothesis of altered hormonal profile, no mechanisms that explain GBM formation or progression in young patients with breast cancer have been identified so far.

Moreover, this case demonstrates once again that brain neoplasia should be considered as a possible cause of mental status alterations during pregnancy or postpartum [1]. To our knowledge, only two cases of brain tumor mimicking postpartum depression have been reported so far [2, 3]. Interestingly, the left frontal lobe was affected in all reported cases [2, 3], including our patient’s, which is in line with a meta-analysis that found a putative (although nonsignificant) association between mood symptoms and tumors located in the frontal lobe [13]. In addition, our report emphasizes the need for a systematic approach for patients with altered mental status, including those with psychiatric presentations [14], inclusive of performing neuroimaging. Thus, practicing psychiatrists must not be deceived by normal physical examinations and blood analyses, because nothing can replace the clinical impression based on a comprehensive psychiatric interview.

## Conclusions

This case report adds to the evidence that postpartum depression may have organic causes in some cases, a fact that needs to be considered in the clinical setting. Profound medical history and psychopathological assessment are fundamental tools. They may alone justify brain imaging in the case of atypical neurocognitive findings, despite normal physical examination and blood analysis results.

## Abbreviations

GBM: Glioblastoma multiforme
IDH1: Isocitrate dehydrogenase 1 mutation
IDH2: Isocitrate dehydrogenase 2 mutation
MRI: Magnetic resonance imaging
PSO: Psychosomatic medicine and psychotherapy
